# A case report of Ovarian hyperstimulation syndrome and corpus luteum rupture in twin pregnancies with IVF-ET

**DOI:** 10.1097/MD.0000000000034238

**Published:** 2023-07-07

**Authors:** Yunliao Luo, Huajuan Shen, Hongjing Li, Zongjian Tan, Chaojun Chen, Weiming Chen, Jun He

**Affiliations:** a Department of Obstetrics and Gynecology, Hezhang County People’s Hospital, Bijie City, Guizhou Province, China; b Department of Reproductive, Guizhou Provincial People’s Hospital, Guiyang City, Guizhou Province, China; c Medical College of Guizhou University, Guiyang City, Guizhou Province, China.

**Keywords:** IVF-ET, luteal rupture, OHSS

## Abstract

Ovarian hyperstimulation syndrome (OHSS) is a common complication during assisted conception treatment, mostly seen in patients with ovarian hyperresponsiveness such as polycystic ovary syndrome, especially in post-invitro fertilization and embryo transfer (IVF-ET) pregnancies. Its main symptoms are abdominal distension, abdominal pain, nausea and vomiting with ascites, pleural fluid, leukocytosis, hemoconcentration and hypercoagulation. This disease is a self-limiting disease and can be gradually cured by rehydration, albumin infusion and correction of electrolyte disorders in moderate to severe cases. Luteal rupture is a more common gynecological emergency abdomen. The combination of twin pregnancy, OHSS and ruptured corpus luteum is very rare. We successfully avoided the stimulation of the risk of pregnancy abortion by surgical exploration through dynamic ultrasound monitoring and vital signs observation in the absence of experience in primary care, and the patient hard-won twin pregnancy was successfully treated conservatively.

**Patient concerns:**

The patient is a 30-year-old post-IVF-ET woman with an established twin pregnancy, OHSS and sudden onset of lower abdominal pain.

**Diagnosis:**

Twin pregnancy, OHSS combined with ruptured corpus luteum.

**Interventions:**

Rehydration, albumin infusion, low molecular heparin for thromboprophylaxis, luteinizing support, ambulatory ultrasound monitoring.

**Outcomes:**

After more than 10 days of standardized treatment for OHSS, dynamic ultrasound monitoring and close observation of vital signs, the patient was discharged cured of her condition and is continuing her pregnancy.

**Conclusion:**

Our case shows that the possibility of acute abdominal rupture of the corpus luteum is still present in the case of combined OHSS in pregnancy, and that some patients with corpus luteum rupture can heal spontaneously during close testing to avoid the increased risk of miscarriage with surgical exploration.

## 1. Introduction

In IVF clinical practice, aspirin and low molecular heparin sodium are commonly used to prevent hypercoagulable states caused by ovarian hyperstimulation syndrome (OHSS) due to the high incidence of OHSS. However, anticoagulants overuse leads to abnormalities in coagulation caused serious consequences in terms of internal bleeding. When luteal rupture occurs in conjunction with OHSS, patient abdominal pain due to excessive ovarian enlargement and the condition of luteal rupture can easily go unnoticed and become a serious threat to the patient life. Therefore, the combination of OHSS with luteal rupture should be taken seriously in clinical practice.

## 2. Case

**Figure 1.**
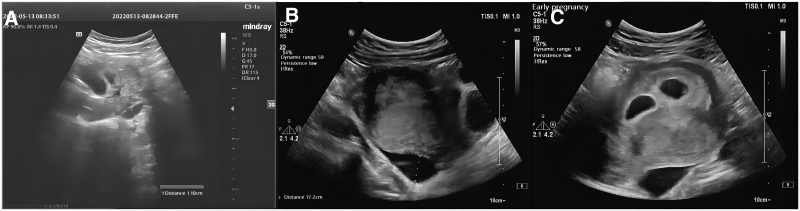
(A) Effusion from left ovary 15 d after admission. hypoechoic mass with 11 mm effusion from left ovary. (B) Pelvic ultrasound 15 d after admission. Pelvic effusion increased to 17.2 mm after 49 min. (C) Ultrasound monitoring of the gestational sac. the size of 21*12 mm dark liquid area at the time of 2 h 51 min.

A 30-year-old female with an abdominal circumference of 76 cm, a weight of 48 kg, G0P0, and abdominal distension and pain 7 days after fresh embryo transfer was admitted to the obstetrics and gynecology department after 10 days of aggravation. The patient assisted reproduction protocol was a long protocol of ovulation promotion and after embryo transfer; she was given conventional luteal support with 10 mg bid. Dydrogesterone tablets orally, 100 mg bid progesterone vaginal suppositories daily, 40 mg/day progesterone injection intramuscularly daily, 4000 IU enoxaparin intramuscularly daily, and 100 mg bid aspirin orally daily. The admission ultrasound suggested 18mm of endometrium, early intrauterine pregnancy, twin chorion and twin lamb pregnancies, bilateral ovarian enlargement (right 61*48 mm, left 62*48 mm) and pelvic fluid depth of 73 mm. After 10 days of admission, hepatic function was impaired (alanine amino transferase = 182 µ/L, aspartate transaminase = 43 µ/L) (Table 1), and enoxaparin was stopped, posterior fornix fluid 27 mm deep. 15 days after admission, sudden onset of severe distension and pressure pain in the left lower abdomen, no muscle tension and rebound pain, accompanied by lumbosacral pain, no anal swelling, vaginal bleeding and abnormal discharge, the rest of the signs were normal; ultrasound suggested normal fetal heart, further enlargement of bilateral ovaries, suggesting multiple cystic echogenicity, pelvic ultrasound suggested about 17 mm liquid dark area, Left ovary 44*30 mm hypoechoic mass with 11 mm effusion (Fig. 1A), pelvic effusion increased to 17.2 mm after 49 minutes (Fig. 1B); meanwhile, routine blood, coagulation mechanism, liver and kidney function were checked, except alanine amino transferase and DD dimer. The ultrasound revealed that the discomfort subsided after 2 hours and that, at the time of 2 hours and fifty minutes, there was a sizeable liquid-dark area adjacent to the sac measuring 21 mm by 12 mm (Fig. 1C). The patient is currently carrying a healthy pregnancy after spending 19 days in the hospital. Current follow-up normal pregnancy after 1 month, 2 month.

**Table 1:** Liver function and coagulation changes.

Time	ALT (U/L)	AST (8–40 U/L)	PROG (nmol/L)	D-Dimer (0.5–1 µg/mL)	PT	PT-INR	APTT	TT
5 d	181↑	85↑	740↑	1.22↑	Normal	Normal	Normal	Normal
10 d	182↑	43↑	/	1.39↑	/	/	/	/
Abdominal pain	88↑	Normal	/	1.06↑	Normal	Normal	Normal	Normal

ALT = alanine amino transferase, AST = aspartate transaminase.

### 2.1. Diagnosis

#### 2.1.1. OHSS and pregnancy.

The patient external history of invitro fertilization and embryo transfer (IVF-ET), abdominal distension, urine human chorionic gonadotropin findings. Due to moderate OHSS, the patient was brought to the gynecology department. Following admission, the patient continued daily rehydration and symptomatic support therapy in accordance with the principles of OHSS treatment, as well as luteinizing support with aspirin 100 mg bid orally and enoxaparin 40 mg/day subcutaneously daily as prescribed by the outside hospital at the patient request. In order to prevent further liver damage, enoxaparin was stopped on the fifth day following admission due to abnormal liver function. As a result, liver function gradually decreased and returned to normal at the release time.

#### 2.1.2. Hemorrhagic corpus luteum.

The patient recently gained acute left lower abdomen distension with lumbosacral pain and considerable left lower abdominal discomfort without myalgias or rebound pain on the fifteenth day following admission. An ultrasound performed at the patient bedside in the emergency room revealed that the left ovary volume, the cystic echogenicity inside of it, and the volume of fluid in the pelvis had all increased significantly relative to before. At first, we could not rule out the possibility of left ovarian torsion, but later dynamic ultrasound observation revealed a poorly transmissible liquid dark area next to the left ovary moving from the left ovarian cystic echogenicity inward to the lateral quicksand-like movement, and no ovarian torsional vascular tip-like echogenicity was seen, so the diagnosis was left ovarian ruptured corpus luteum bleeding. The patient abdominal pain improved significantly after 2 hours, after which the symptoms gradually disappeared and the pelvic fluid disappeared at the time of discharge.

## 3. Discussion

The incidence of OHSS is approximately 1% to 5%^[1]^ and can be as high as 30% in IVF-ET,^[2]^ and the mechanism of occurrence remains unknown.^[3]^ In this case, the diagnosis was confirmed by symptoms and imaging after admission. Aspirin^[4,5]^ and low-molecular heparin have been reported to increase the rate of embryo implantation,^[6]^ so the patient was treated with both enoxaparin and aspirin anticoagulants at an outside hospital.

The incidence of combined luteal rupture in pregnancy is about 13% of the pregnancy population, and the combination of OHSS and luteal rupture in twin pregnancies after IVF-ET is rarely reported and can be confused with a variety of gynecological emergencies.^[7,8]^

This case should alert us to antagonist regimens and appropriate Gn initiation doses^[9]^ should be chosen as much as possible in patients with ovarian hyper-responsiveness; the indications and dosages for the use of aspirin and low molecular heparin in IVF-ET should be strictly controlled^[10,11]^; luteinizing support in IVF-ET should be avoided in multiple species overdose^[12,13]^; anticoagulants should be discontinued promptly after the occurrence of bleeding disorders; after the occurrence of luteal rupture, conservative treatment can be taken for a certain period of time when the patient vital signs are stable and the disease is self-limiting in some patients.^[14]^

## 4. Conclusion

Pay more attention to clinical practice for OHSS patient, especially for abdominal pain and pay attention to OHSS with transvaginal ultrasound for pelvic. Make the right treatment decision when the disease condition changes rapidly in a short time.

## Acknowledgments

Thanks the patient supports this observation.

## Author contributions

**Conceptualization:** Zongjian Tan.

**Investigation:** Huajuan Shen.

**Methodology:** Weiming Chen.

**Supervision:** Chaojun Chen, Weiming Chen.

**Validation:** Hongjing Li.

**Writing – original draft:** Yunliao Luo.

**Writing – review & editing:** Jun He.
